# Influence of diabetes on the risk of deep vein thrombosis of patients after total knee arthroplasty: a meta-analysis

**DOI:** 10.1186/s13018-024-04624-z

**Published:** 2024-03-04

**Authors:** Jingzhi An, Li Han, Xiaojuan Ma, Yanyan Chang, Cuixin Zhang

**Affiliations:** 1 Department of Clinical Pharmacy, The Third Hospital of Hebei Medical University, 139 Ziqiang Road, Qiaoxi District, Shijiazhuang, 050051 China; 2 Graduate School, Hebei Medical University, Shijiazhuang, 050017 China

**Keywords:** Total knee arthroplasty, Deep vein thrombosis, Diabetes, Risk factor, Meta-analysis

## Abstract

**Background:**

Previous studies evaluating the influence of diabetes on the risk of deep vein thrombosis (DVT) after total knee arthroplasty (TKA) showed inconsistent results. The aim of the study was to systematically evaluate the association between diabetes and DVT after TKA in a meta-analysis.

**Methods:**

An extensive search was conducted in PubMed, Embase, and Web of Science to identify relevant cohort studies. Random-effects models were employed to pool the results after taking account of the potential influence of heterogeneity.

**Results:**

Thirteen cohort studies involving 546,156 patients receiving TKA were included, with 71,110 (13.0%) diabetic patients before surgery and 1479 (2.1%) patients diagnosed as DVT after surgery. Overall, diabetes was associated with an increased risk of DVT after TKA (risk ratio [RR]: 1.43, 95% confidence interval [CI]: 1.12–1.84, *p* = 0.004; *I*^2^ = 44%). Sensitivity analysis limited to studies with chemoprophylaxis (RR: 1.96, 95% CI: 1.50–2.54), and studies with multivariate analysis (RR: 1.54, 95% CI: 1.12–2.11) showed consistent results. Subgroup analysis showed that diabetes was associated with higher risk of postoperative DVT in Asian countries (RR: 1.93, 95% CI: 1.49–2.52, *p* < 0.001; *I*^2^ = 1%) but not in Western countries (RR: 1.07, 95% CI: 0.86–1.34, *p* = 0.52; *I*^2^ = 0%; *p* for subgroup difference < 0.001).

**Conclusion:**

Diabetes may be a risk factor for DVT after TKA, even with the chemoprophylaxis of anticoagulants. The association between diabetes and DVT after TKA may be more remarkable in patients from Asian countries.

## Introduction

Diabetes is a common metabolic disorder characterized by hyperglycemia resulted from insufficient pancreatic islet β cell function and insulin resistance [1]. With the aging of the globe population, the prevalence of diabetes is expected to continuously increase in the future decades [2, 3]. Patients with diabetes are shown to be associated with higher risks of cardiovascular complications [4]. Moreover, increasing evidence suggests that for patients undergoing surgical procedures, diabetes may also be associated with poor postoperative outcomes [5, 6].

Total knee arthroplasty (TKA) is a frequently performed orthopedic surgery primarily for the treatment of severe knee joint diseases [7, 8]. Also due to the aging of the global population, TKA is increasingly performed in the recent years and patients receiving TKA are also expected to continuously grow in the future decades [9]. Deep vein thrombosis (DVT) is one of the common and severe postoperative complications in patients receiving TKA [10]. Clinically, DVT may cause pulmonary embolism, leading to increased morbidity and mortality in these patients [11, 12]. Therefore, identification of risk factors for DVT after TKA is important for early identification of high-risk patients. It is reported that up to 50% of patients receiving TKA may have diabetes before the surgery [13]. Some previous studies suggested that diabetes may be a risk factor for DVT after TKA [14–16], while a few other studies did not show the same results [17–21]. In view of this inconsistency, we performed a systematic review and meta-analysis to comprehensively evaluate the association between diabetes and DVT after TKA. In recent years, continuously efforts have been made for preventing DVT after TKA, such as use of chemoprophylaxis anticoagulants [22, 23]. Specifically, we aimed to evaluate if the potential association between diabetes and DVT after TKA remains even with the chemoprophylaxis of anticoagulants.

## Materials and methods

The research followed the Meta-analyses Of Observational Studies in Epidemiology (MOOSE) guideline [24] and the Cochrane Handbook [25] consistently during the phase of planning, execution, and documentation.

### Inclusion and exclusion criteria of studies

The development of inclusion criteria adhered to the PICOS recommendations and aligned with the objective of the meta-analysis.

P (patients): Adult patients (18 years or older) undergoing TKA.

I (exposure): Diabetes (type 1 or type 2 diabetes) was diagnosed and considered as the exposure before surgery.

C (control): Patients without the diagnosis of diabetes before surgery were considered as control.

O (outcomes): The primary outcome of the meta-analysis was the incidence of DVT after TKA, compared between patients with diabetes and normoglycemia.

S (study design): Cohort studies, including prospective and retrospective cohorts.

Excluded from the meta-analysis were literature reviews, editorials, meta-analyses, and studies that include patients not undergoing TKA, did not evaluate diabetes as exposure, or did not report the outcomes of interest. In instances where there was a duplication of patient populations, the study with the most extensive sample size was incorporated into the meta-analysis.

### Search of databases

Studies relevant to the objective of the meta-analysis were identified by search of electronic databases, namely PubMed, Embase, and Web of Science encompassing the period from inception to November 10, 2023. The search strategy employed relevant terms pertaining to the subject matter of our investigation, aiming to identify studies published within this timeframe, which included: (1) "diabetes" OR "diabetic"; (2) "total knee replacement" OR "total knee arthroplasty"; and (3) "deep vein thrombosis" OR "DVT." Only studies that met the criteria of being published as full-length articles in English and appearing in peer-reviewed journals were included in our analysis. Additionally, during our manual screening process, we thoroughly examined the references cited in relevant original and review articles to identify any potentially relevant studies.

### Data extraction and quality evaluation

Two authors conducted literature searches, collected data, and assessed the quality of the studies separately. In instances where inconsistencies arose, the authors engaged in discussions to reach a consensus. The analysis of the studies involved gathering data pertaining to study details, design attributes, sample size, patient demographics, definition of diabetes, number of patients with diabetes before surgery, follow-up duration, methods for DVT prevention, methods for detection of DVT after TKA, number of patients who developed DVT after TKA, and variables adjusted when the association between diabetes and postoperative DVT was reported. The quality of the study was evaluated using the Newcastle–Ottawa Scale (NOS) [26]. This scale assesses the quality of cohort studies based on three dimensions: the selection of study groups, the comparability of these groups, and the ascertainment of the outcome of interest. The NOS varied between one and nine stars, with a higher star indicating a better study quality.

### Statistics

Odds ratio (ORs) and their corresponding 95% confidence intervals (CIs) were utilized as the variables to assess the relationship between diabetes and the risk of DVT after TKA. In order to stabilize and standardize the variance, a logarithmic transformation was implemented on the OR and its corresponding standard error in each study [27]. The Cochrane Q test and the *I*^2^ statistic [28] were utilized to assess between-study heterogeneity. A value of *I*^2^ exceeding 50% signifies the existence of substantial heterogeneity among the studies. The random-effects model was employed for synthesizing the results, as it is acknowledged for its ability to accommodate potential heterogeneity [25]. A sensitivity analysis by excluding one study at a time was performed to evaluate the robustness of the findings [25]. Moreover, sensitivity analyses limiting to studies with the use of chemoprophylaxis for DVT and to studies with multivariate analyses only were also performed. In addition, subgroup analyses according to the study country (Asian or Western) and NOS were also performed. Publication bias was estimated using a funnel plot, which involved visual assessments of symmetry, as well as Egger's regression asymmetry test [29]. The statistical analyses were conducted using RevMan (version 5.1; Cochrane Collaboration, Oxford, UK) and Stata software (version 12.0; Stata Corporation, College Station, TX).

## Results

### Database search and study retrieval

**Fig. 1:**
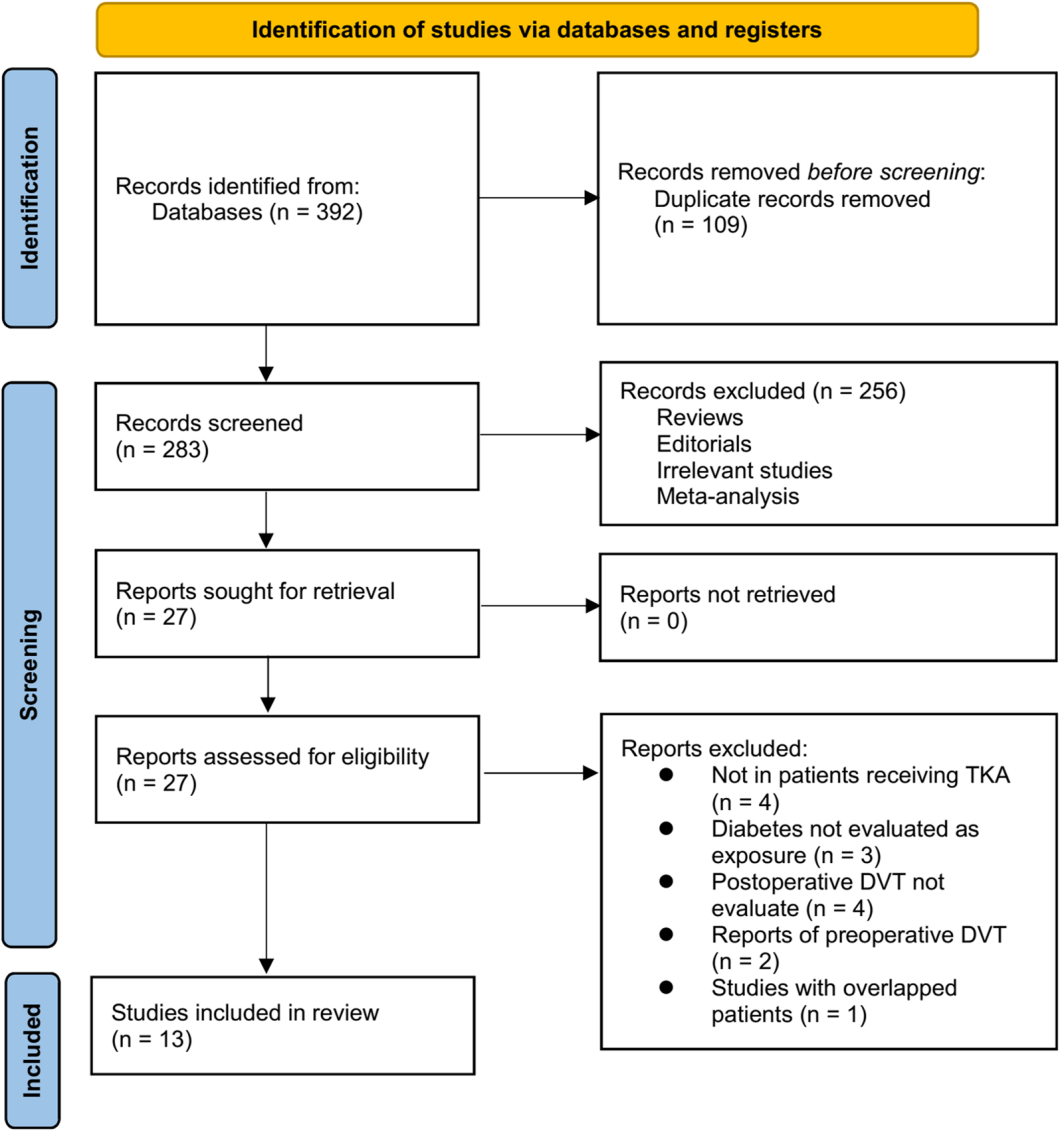
Flowchart of database search and study inclusion;

Figure 1 illustrates the procedure employed for conducting the literature search and study retrieval. Initially, a total of 392 records were acquired from the designated database, and subsequently, 109 duplicate entries were eliminated. Upon scrutinizing the titles and abstracts, an additional 256 studies were excluded due to their incompatibility with the objectives of the meta-analysis. Following comprehensive evaluations of the full texts of 27 studies, 14 were excluded based on the rationales outlined in Fig. 1. Consequently, thirteen studies [14–21, 30–34] were deemed suitable for the subsequent meta-analysis.

### Study characteristics

Overall, 13 retrospective cohort studies [14–21, 30–34] were included in the meta-analysis. The characteristics of the studies and the included patients are shown in Table 1. These studies were published between 2003 and 2023 and performed in the USA, Spain, China, and Korea. Overall, 546,156 patients receiving TKA were included. The mean ages of the patients were 66 to 77 years, and the proportions of men were 9% to 41%. All of the included studies observed the influence of diabetes on postoperative DVT except for one study [33], which investigated the influence of type 2 diabetes (T2D) only. Accordingly, 71,110 (13.0%) patients were with diabetes before surgery. The follow-up durations varied from during hospitalization to 90 days after surgery. Chemoprophylaxis for DVT with anticoagulants including heparin, low molecular weight heparin, or rivaroxaban was reported in eight studies [14–17, 20, 31, 32, 34], not used in one study [18], and not reported in another four studies [19, 21, 30, 33]. Confirmation of postoperative DVT was performed with clinical screening via Doppler ultrasonography or lower limb venography in 11 studies [14–21, 31, 32, 34] and evidenced by the International Classification of Disease codes in two studies [30, 33]. Accordingly, 1479 (2.1%) patients were diagnosed as DVT after surgery. Multivariate analyses were performed in ten studies when the association between diabetes and DVT after TKA was estimated [14–19, 30, 31, 33, 34], which adjusted the potential confounding factors such as age, sex, body mass index, and comorbidities. In the other three studies [20, 21, 32], univariate analyses were performed. The NOS of these studies ranged from six to nine, indicating moderate to good study quality (Table 2).

**Table 1:** Characteristics of the included studies

Study	Country	Design	Number of patients	Mean age (years)	Men (%)	Definition of DM	Number of patients with DM	Follow-up duration	Methods for DVT prevention	Methods for confirmation of postoperative DVT	Number of patients with postoperative DVT	Variables adjusted
Meding 2003	US	RC	3519	70	40.7	T1D or T2D	291	During hospitalization	IV heparin during surgery	Clinically screened with DUS for all the patients	13	Age and sex
Bolognesi 2008	US	RC	458,986	67.9	38.8	T1D or T2D	46,315	During hospitalization	NR	ICD codes	225	Age, race, sex, and income
Moon 2008	Korea	RC	342	67.5	9.4	T1D or T2D	171	During hospitalization	Not performed	Clinically screened with DUS for all the patients	5	Age, sex, and BMI
Wang 2013	China	RC	245	67	29.4	T1D or T2D	53	14 days	LMWH for 14 days starting on the first postoperative day	Clinically screened with DUS for all the patients	125	Age, sex, type of anesthesia, hypertension, and CAD
Adams 2013	US	RC	40,491	68	37.5	T1D or T2D	7567	90 days	NR	Clinically screened with DUS for all the patients	200	Age, sex, BMI, and CCI
Zhao 2014	China	RC	358	68	33.5	T1D or T2D	70	14 days	LMWH for 14 days starting on the first postoperative day	Clinically screened with DUS for all the patients	198	Age, sex, and hypertension
Kang 2015	China	RC	1025	76.8	36.7	T1D or T2D	260	During hospitalization	LMWH postoperative	Clinically screened with DUS for all the patients	175	Age, sex, BMI, serum Hcy, FBG, surgery time, and use of foot pump
Song 2016	China	RC	109	66.8	18.3	T1D or T2D	14	During hospitalization	Rivaroxaban or LMWH postoperative	Clinically diagnosed with lower limb venography	26	None
Martinez 2017	Spain	RC	26,640	71.6	32.3	T2DM	13,320	During hospitalization	NR	ICD codes	67	Age, sex, and comorbidities
Dai 2020	China	RC	431	67.9	19	T1D or T2D	70	During hospitalization	LMWH postoperative	Clinically screened with DUS for all the patients	95	None
Gu 2020	US	RC	13,246	NR	40.1	T1D or T2D	2865	30 days	NR	Clinically screened with DUS for all the patients	121	None
Lee 2021	Korea	RC	103	NR	11	T1D or T2D	23	During hospitalization	LMWH postoperative	Clinically diagnosed with lower limb venography	22	Age, sex, type of anesthesia, and BMI
Gao 2023	China	RC	661	69.8	32.5	T1D or T2D	91	During hospitalization	LMWH postoperative	Clinically diagnosed with lower limb venography	207	Age, sex, BMI, smoking, comorbidities, surgery time, and type of anesthesia

*DM* diabetes mellitus; *DVT* deep vein thrombosis; *RC* retrospective cohort; *T1D* type 1 diabetes; *T2D* type 2 diabetes; *NR* not reported; *LMWH* low molecular weight heparin; *DUS* Doppler ultrasonography; *ICD* International Classification of Disease; *CAD* coronary artery disease; *BMI* body mass index; *Hcy* homocysteine; *FBG* fasting blood glucose; *CCI* Charlson comorbidity index

**Table 2:** Study quality evaluation via the Newcastle–Ottawa Scale

Study	Representativeness of the exposed cohort	Selection of the non-exposed cohort	Ascertainment of exposure	Outcome not present at baseline	Control for age and sex	Control for other confounding factors	Assessment of outcome	Enough long follow-up duration	Adequacy of follow-up of cohorts	Total
Meding 2003	0	1	1	1	1	0	1	1	1	7
Bolognesi 2008	1	1	1	1	1	0	0	1	1	7
Moon 2008	0	1	1	1	1	1	1	1	1	8
Wang 2013	0	1	1	1	1	1	1	1	1	8
Adams 2013	0	1	1	1	1	1	1	1	1	8
Zhao 2014	0	1	1	1	1	0	1	1	1	7
Kang 2015	0	1	1	1	1	1	1	1	1	8
Song 2016	1	1	1	1	0	0	1	1	1	7
Martinez 2017	0	1	1	1	1	1	1	1	1	8
Dai 2020	1	1	1	1	0	0	1	1	1	7
Gu 2020	0	1	1	1	0	0	1	1	1	6
Lee 2021	0	1	1	1	1	1	1	1	1	8
Gao 2023	1	1	1	1	1	1	1	1	1	9

### Meta-analysis results

**Fig. 2:**
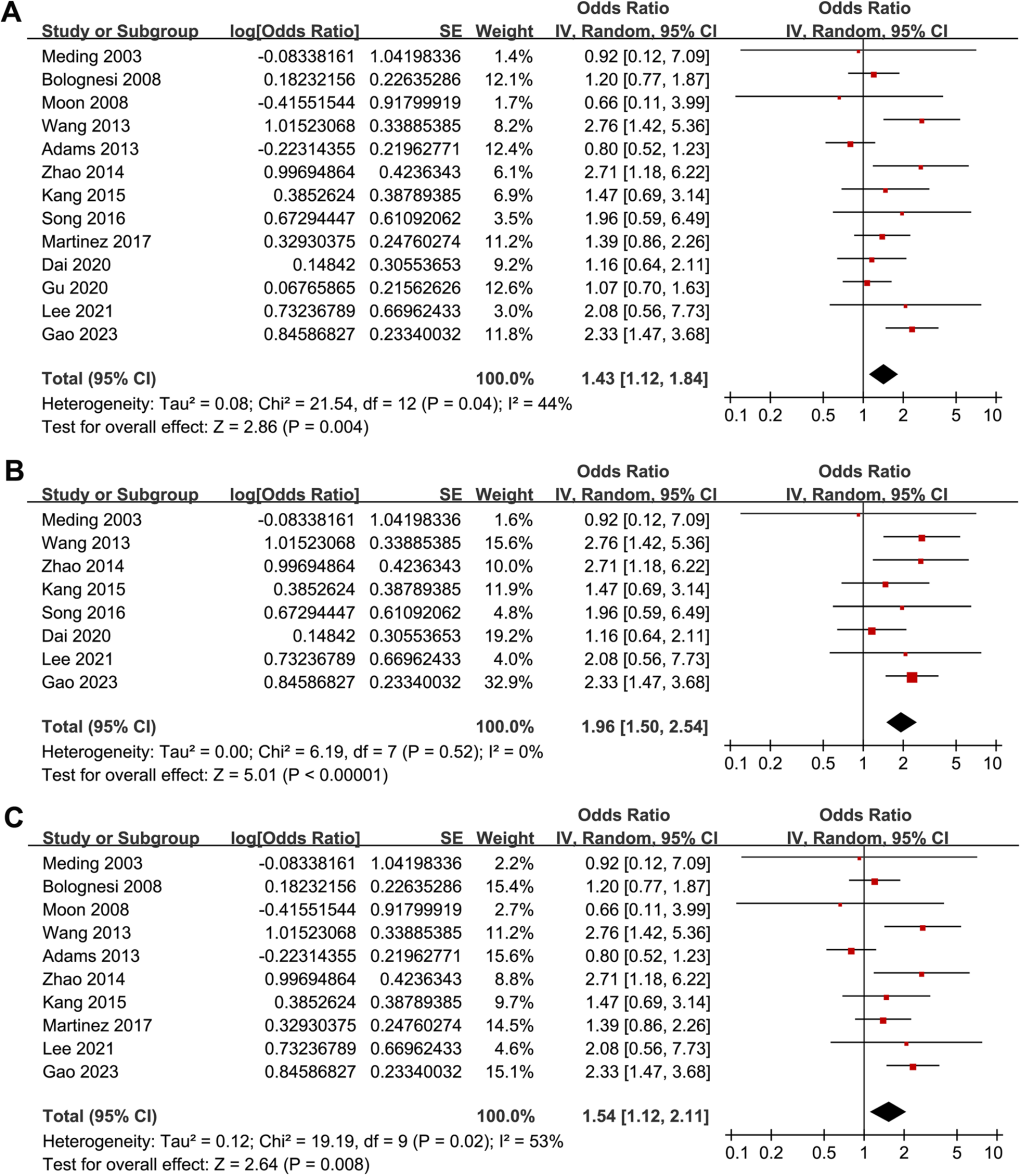
Forest plots for the meta-analysis regarding the association between diabetes and DVT after TKA; A, forest plots for the overall meta-analysis; B, forest plots for the sensitivity analysis limited to studies with chemoprophylaxis for DVT; and C, forest plots for the sensitivity analysis limited to studies with multivariate analyses;

**Fig. 3:**
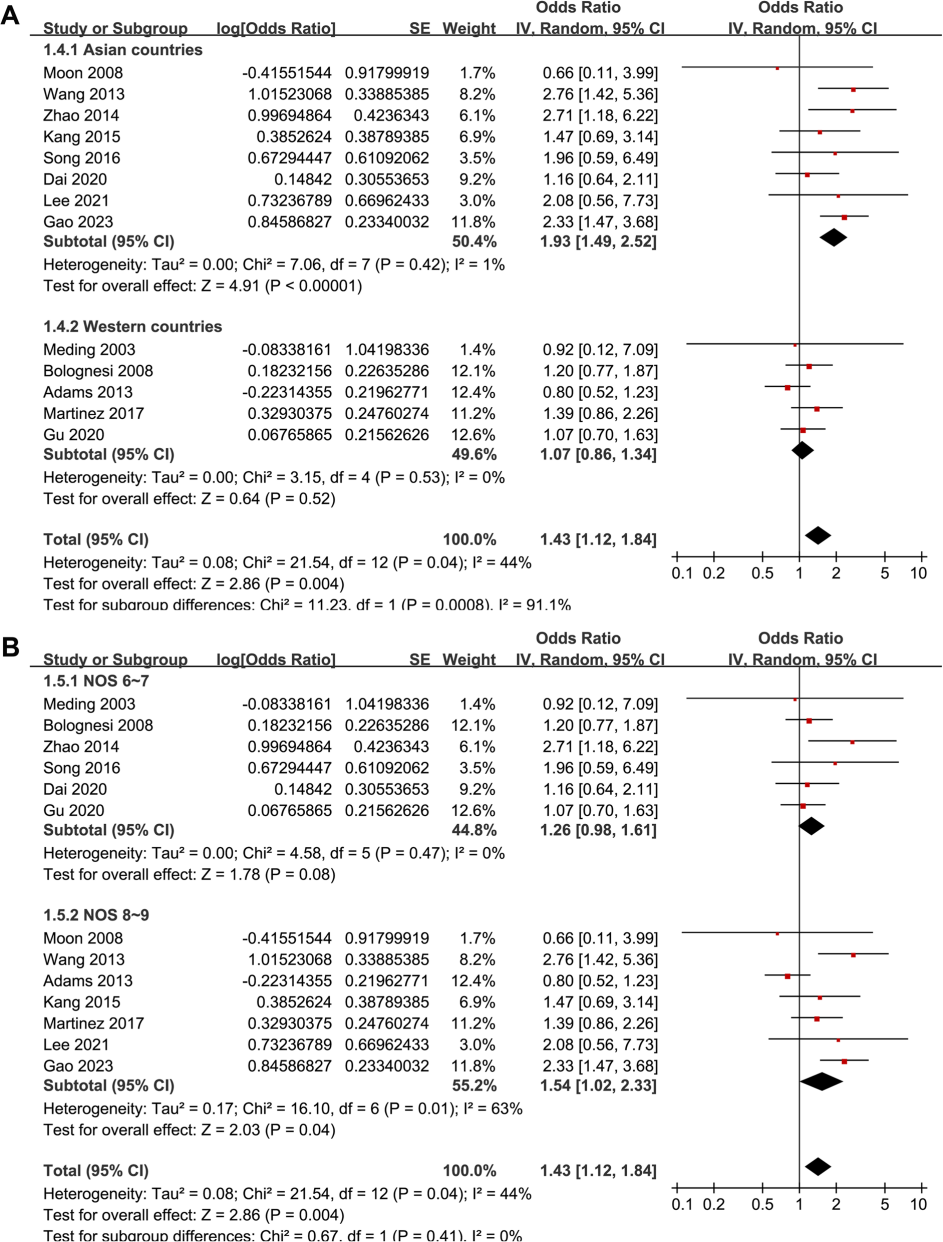
Forest plots for the subgroup analyses regarding the association between diabetes and DVT after TKA; A, forest plots for the subgroup analyses according to the study country; and B, forest plots for the subgroup analyses according to the study quality score;

Pooled results with 13 studies showed that overall, diabetes was associated with an increased risk of DVT after TKA (RR: 1.43, 95% CI: 1.12–1.84, *p* = 0.004; Fig. 2A) with moderate heterogeneity (*I*^2^ = 44%). Sensitivity analyses by excluding one study at a time showed similar results (data not shown). In addition, sensitivity analysis limited to studies with chemoprophylaxis for DVT (RR: 1.96, 95% CI: 1.50–2.54, *p* < 0.001; *I*^2^ = 0%; Fig. 2B) and studies with multivariate analysis (RR: 1.54, 95% CI: 1.12–2.11; *p* = 0.008; *I*^2^ = 53%; Fig. 2C) also showed consistent results. For most of the included studies, study country rather than the ethnicity of the patients was reported. Accordingly, we have performed subgroup analysis according to the country of the study (Asian countries versus western countries), which may somewhat reflect the influence of patient ethnicity on the outcome. It was shown that that diabetes was associated with higher risk of postoperative DVT in Asian countries (RR: 1.93, 95% CI: 1.49–2.52, *p* < 0.001; *I*^2^ = 1%) but not in Western countries (RR: 1.07, 95% CI: 0.86–1.34, *p* = 0.52; *I*^2^ = 0%; p for subgroup difference < 0.001; Fig. 3A). Subgroup analysis according to NOS showed similar results (*p* for subgroup difference = 0.41; Fig. 3B).

### Publication bias

**Fig. 4:**
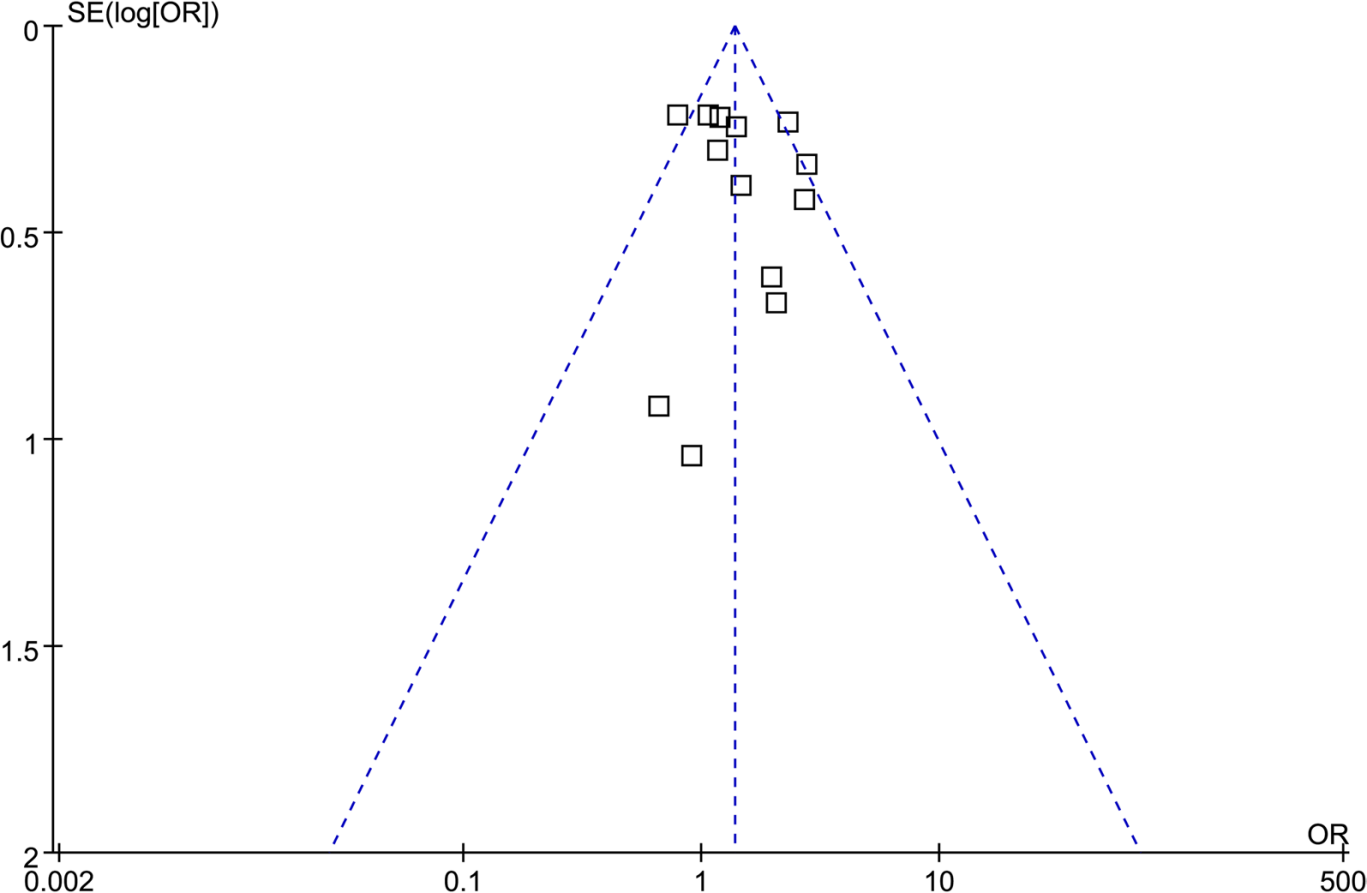
Funnel plots for the publication biases underlying the meta-analyses of the association between diabetes and DVT after TKA

The funnel plots depicting the meta-analyses of the association between diabetes and DVT after TKA are shown in Fig. 4. Upon visual inspection, the plots exhibit symmetrical patterns, indicating a minimal presence of publication bias. The Egger’s regression test also suggested a low risk of publication bias (*p* = 0.42).

## Discussion

This meta-analysis synthesized the results of 13 availably cohort studies, and the results showed that compared to patients with normoglycemia, patients with diabetes before the surgery had a higher incidence of DVT after TKA. Further sensitivity analyses showed consistent results in patients with chemoprophylaxis for DVT and in studies with multivariate analyses after adjustment of potential confounding factors. Moreover, subgroup analysis suggested that diabetes was associated with a higher risk of DVT after KTA in studies from Asian countries, but not in those from western countries. Taken together, results of the meta-analysis indicate that diabetes may be a risk factor for DVT after TKA, even with the chemoprophylaxis of anticoagulants. The association between diabetes and DVT after TKA may be more remarkable in patients from Asian countries.

To the best of our knowledge, two previous meta-analyses have evaluated the influence of diabetes on DVT after TKA. One early meta-analysis published in 2014 included six available studies and showed that diabetes may increase the risk of DVT after TKA. Another meta-analysis published in 2015 included the same studies, which also retrieved similar results. However, significant heterogeneity was observed among these meta-analyses. Due to the limited number of available datasets, the authors did not perform further analyses to identify the source of heterogeneity. Moreover, chemoprophylaxis with anticoagulants has been suggested to be effective in reducing the risk of DVT after TKA [35]. However, none of the meta-analyses observed the potential influence of chemoprophylaxis on the association. In our meta-analysis, an extensive literature search was performed in three commonly used electronic databases, which retrieved 13 up-to-date cohort studies according to the aim of the meta-analysis. Only cohort studies were considered in this meta-analysis, thereby providing a longitudinal relationship between prediabetes and postoperative DVT. Subsequent sensitivity meta-analysis by omitting one study at a time showed consistent results, further reflecting the robustness of the finding. More important, sensitivity analysis limited to studies with the use of chemoprophylaxis for DVT showed consistent results, which support an association between diabetes and increased risk of DVT after TKA, even in patients who received chemoprophylaxis. Also, sensitivity analysis limited to studies with multivariate analysis suggested a similar association, which indicates that the association between diabetes and increased risk of DVT after TKA was independent of the potential confounding factors, such as age. This is important because aging has been related to both diabetes [36] and postoperative DVT [37]. Finally, subgroup analysis according to study country showed that diabetes was associated with higher risk of postoperative DVT in studies from Asian countries, but not in Western countries. These findings suggest that ethnicity may affect the association between diabetes and risk of DVT after TKA, which may fully explain the source of heterogeneity. Taken together, these findings support that the preexisting diabetes may be a risk factor for postoperative DVT of patients after TKA.

The mechanisms underlying the association between diabetes and DVT after TKA remain unknown. Pathophysiologically, diabetes is related to chronic low-degree inflammation [38], oxidative stress response [39], endothelial dysfunction [40], and activated coagulative system [41], which are all likely to be involved in the pathogenesis of postoperative DVT. In addition, although we found that ethnicity of the patients may affect the association between diabetes and DVT after TKA, the reasons for the finding are also unknown. Previous studies have showed that the incidence of DVT of Asian patients after TKA is lower than patients other ethnicities [42]. The mechanisms underlying the influence of ethnicity on the association between preexisting diabetes and postoperative DVT should be determined in the future.

This meta-analysis has limitations. First, all of the included studies were of retrospective design, which may expose the studies to the influences of selection and recall biases. Second, we were unable to determine if T1D and T2D have similar influence on the risk of DVT after TKA. Moreover, for sensitivity analysis limited to studies with chemoprophylaxis, none of the included studies used new oral anticoagulants (NOAC). It is important to determine if the association between diabetes and postoperative DVT remains in patients received NOAC for DVT prophylaxis. In addition, although sensitivity analysis limited to studies with multivariate analysis showed consistent results, we could not exclude the possibility of other residual factors which may confound the association between diabetes and postoperative DVT, such as the differences of antidiabetic treatments. Finally, this meta-analysis was based on observational studies, which could not determine a causative relationship between preexisting diabetes and the risk of DVT after TKA. It has to be mentioned that the above limitations were inherited to the design of meta-analysis of observational studies and meta-analysis based on study-level data rather than individual patient data, and accordingly, it is unable to further address the above limitations at current stage. Future prospective and clinical trials are warranted to address the above potential limitations.

## Conclusions

In conclusion, results of the meta-analysis indicate that compared to patients with normoglycemia, patients with diabetes before the surgery had a higher incidence of DVT after TKA, even in patients with the chemoprophylaxis of anticoagulants. The association between diabetes and DVT after TKA may be more remarkable in patients from Asian countries. Although these findings should be validated in large-scale prospective studies, results of the meta-analysis support that diabetes may be a risk factor for DVT in patients receiving TKA.

## Data Availability

All data generated or analyzed during this study are included in this article.
